# Targeting the NF-κB pathway as a potential regulator of immune checkpoints in cancer immunotherapy

**DOI:** 10.1007/s00018-023-05098-8

**Published:** 2024-02-29

**Authors:** Nasim Ebrahimi, Al-Hasnawi Rasool Riyadh Abdulwahid, Atena Mansouri, Nasrin Karimi, Rashid Jafardoust Bostani, Sheida Beiranvand, Samaneh Adelian, Roya Khorram, Reza Vafadar, Michael R. Hamblin, Amir Reza Aref

**Affiliations:** 1 Genetics Division, Department of Cell and Molecular Biology and Microbiology, Faculty of Science and Technology, University of Isfahan, Isfahan, Iran; 2 Medical Campus, Xi’an Jiaotong University, Xi’an, Shaanxi Province China; 3 Department of Biology, Science and Research Branch, Islamic Azad University, Tehran, Iran; 4 Department of Biology, Faculty of Basic Science, Islamic Azad University Damghan Branch, Damghan, Iran; 5 Department of Biology, Urmia Branch, Islamic Azad University, Urmia, Iran; 6 Department of Biology, Faculty of Basic Sciences, Shahrekord Branch, Islamic Azad University, Shahrekord, Iran; 7 Cellular and Molecular Research Center, Basic Health Sciences Institute, Shahrekord University of Medical Sciences, Shahrekord, Iran; 8 Bone and Joint Diseases Research Center, Department of Orthopedic Surgery, Shiraz University of Medical Sciences, Shiraz, Iran; 9 Department of Orthopeadic Surgery, Kerman University of Medical Sciences, Kerman, Iran; 10 Laser Research Centre, Faculty of Health Science, University of Johannesburg, Doornfontein, 2028 South Africa; 11 Radiation Biology Research Center, Iran University of Medical Sciences, Tehran, Iran; 12 Xsphera Biosciences, Translational Medicine Group, 6 Tide Street, Boston, MA 02210 USA; 13 Department of Medical Oncology, Dana-Farber Cancer Institute, Harvard Medical School, Boston, MA 02115 USA

**Keywords:** Immune-related adverse events, Immune checkpoint inhibitors, Regulatory T cells, Immunooncology, Receptor activator of nuclear factor kappa-Β ligand

## Abstract

Advances in cancer immunotherapy over the last decade have led to the development of several agents that affect immune checkpoints. Inhibitory receptors expressed on T cells that negatively regulate the immune response include cytotoxic T‑lymphocyte antigen 4 (CTLA4) and programmed cell death protein 1 (PD1), which have been studied more than similar receptors. Inhibition of these proteins and other immune checkpoints can stimulate the immune system to attack cancer cells, and prevent the tumor from escaping the immune response. However, the administration of anti-PD1 and anti-CTLA4 antibodies has been associated with adverse inflammatory responses similar to autoimmune diseases. The current review discussed the role of the NF-κB pathway as a tumor promoter, and how it can govern inflammatory responses and affect various immune checkpoints. More precise knowledge about the communication between immune checkpoints and NF-κB pathways could increase the effectiveness of immunotherapy and reduce the adverse effects of checkpoint inhibitor therapy.

**Graphical abstract:**

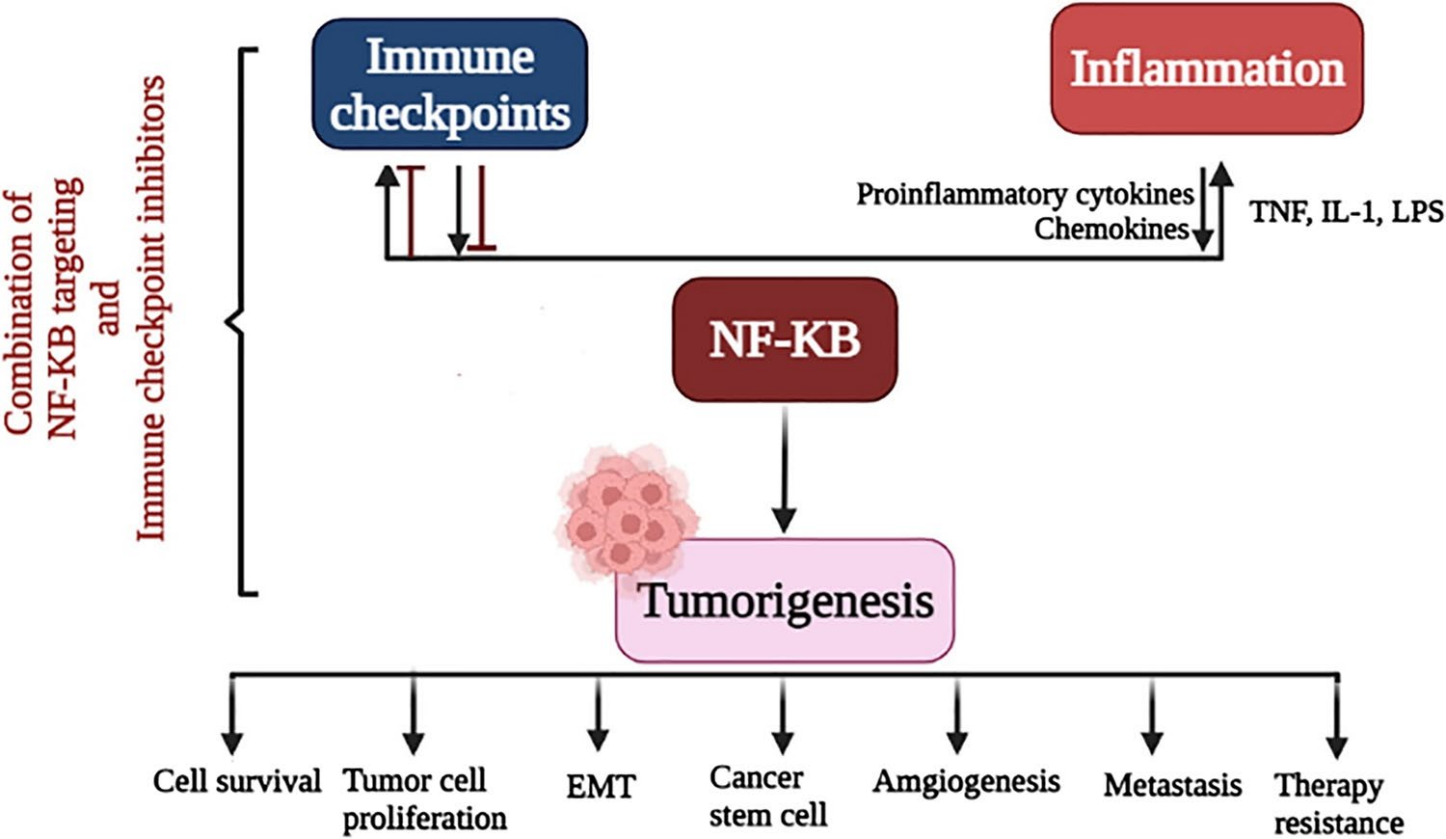

## Introduction

In recent years, novel cancer immunotherapy approaches have been developed, and have made major progress in the fight against cancer. However, the immune system behaves like a double-edged sword, not only protecting the body against foreign agents, but can also attack self-antigens and cause autoimmune diseases. For this reason, the human immune system has built-in mechanisms designed to prevent excessive immune responses against host cells. These mechanisms are called immune checkpoints, and their inhibition by monoclonal antibodies has revolutionized oncoimmunology in recent years [1]. According to some theories, the immune system has evolved not only to combat infections, but also as a way for multicellular organisms to prevent the development of tumors and inhibit the growth of malignant cells [2]. As a response to this attack, cancer cells have developed solutions that allow them to escape from the immune system, and to migrate to distant tissues in the process of metastasis [3]. Activating immune checkpoints to suppress the immune response is considered as an escape mechanisms by which cancer cells can slip from immune system [4]. Novel immune-based therapies are designed to blockade immune checkpoints, and maintain the activity of the immune response against tumor cells [5]. It is known that cancer cells can stimulate specific immune responses against cancer-specific antigens and neoantigens [6]. Although several antibody-based immune checkpoint inhibitors (ICIs) have been investigated in animal studies and clinical trials in recent years [7–10], they are often associated with major side effects and immune-related adverse events (IRAEs) [11, 12]. In this regard, different targets can still be pursued, including the signaling pathways that activate immune checkpoints. Since these pathways are active early in the development of immune checkpoints [13], they may be promising targets for novel immunotherapy.

Members of the nuclear factor-kappa B (NF-κB) family are a group of transcription factors whose primary function governs a wide range of biological processes. Growing evidence has shown the role of NF-κB as an oncogene in various cancers [14]. Over-activation of this pathway has been identified in numerous human malignancies. Recent studies have concentrated on elucidating the effects of NF-κB and its signaling mechanisms in regulating immune responses and inflammation, and have made it an appealing therapeutic target in various cancer immunotherapy studies [15]. The NF-κB pathway contributes in two separate cancer-associated mechanisms. One is concerned with abnormal cell division and cell survival, while the other is concerned with escape from the immune response and the activation of immune checkpoints. In this review article, the potential of the NF-κB pathway as a target in immunotherapy approaches is discussed, and its role in regulating immune checkpoints is analyzed.

## NF-κB under a magnifying glass

The NF-κB protein was discovered about 30 years ago in the nuclei of B lymphocytes by Sen and Baltimore [16]. They realized that NF-κB binds to a specific DNA sequence, 5′-GGGRNWYYCC-3′. They first named this transcription factor based on the gene that was affected and the cell in which it was first identified: nuclear factor that binds close to the antibody κ light-chain gene in B cells [16]. When the role of NF-κB in stimulating the maturation of B cells was discovered, it was realized that it is involved in many immune and cellular processes, including inflammation [15]. However, the role of NF-κB is not solely limited to the immune system, and it is now recognized that many cellular and pathological processes are dependent on NF-κB activity [17]. These roles include the following; (1) immediately after the cell is exposed to an inflammatory stimulus or other stressful condition, a wide range of cellular reactions are activated by NF-κB. Once the inflammatory stimulus is neutralized, the cell returns to a latent state. (2) When invasive stimuli are transient, innate immune cells can defend the body. If the inflammation or invasion is more prolonged, the adaptive immune system is activated by the function of B and T cells involving the strong mediation of NF-κB [17].

It soon became apparent that NF-κB was not a single transcription factor, but a family of interconnected protein complexes. The subunits of this complex cooperate together as hetero-homodimers, and the 5 monomers can produce up to 15 different NF-κB complexes [18]. The main reason for the broad ability of NF-κB to affect cell biology can be traced to its ability to regulate the expression of hundreds of target genes that have the appropriate response element. Surprisingly, each regulatory role for each target gene requires a specific concentration of each component of the NF-κB complex [18]. Specific sequences in the relevant gene bind to NF-κB as a transcription factor, and may cooperate with other specific transcription factors for each gene. Moreover, genetic information affects the NF-κB structure, the half-life of the induced mRNA, and the rate at which the NF-κB binds to the induced pre-mRNA [19].

Five distinct proteins, P100, P105, C-Rel, RelB, and RelA make up the NF-κB transcription factor family [20]. The Rel homology domain (RHD) is an essential domain to bind DNA, to dimerise, and interact with IκB inhibitors, and the three proteins c-Rel, Rel-A (p65), and Rel-B, possess a transcription activating domain (TAD) [21]. P100 (NFKB2) and P105 (NFKB1) are the main precursors of the mature NF-κB transcription factor, which after proteolysis, produce two subunits, P50 (NF-B1) and P52 (NF-B2) [22–24]. As a result, NF-κB is a generic designation that refers to a group of dimeric proteins created by various subunits. p50/RelA is the most studied NF-κB dimer and expressed in various range of cells [25]. The biology and structure of NF-κB has been well reviewed in reference [14].

NF-κB proteins are involved in cellular homeostasis, due to their effect on the transcription of numerous genes [26, 27], including cytokines and Chemokines (IL-6, TNF-alpha), adhesion molecules (intercellular adhesion molecule-1 (ICAM-1), and enzymes and mediators (cyclooxygenase-2 (COX-2), inducible nitric oxide synthase (iNOS)). This is why NF-κB proteins can affect various processes in cells such as, immunity, cell proliferation, inflammation, survival, and even cell death [28]. The roles NF-κB family members in the immune response and adjustment of inflammation on one hand, and on the other hand, their involvement in cell division and death, especially in cancer, puts these proteins in an ideal position for cancer immunotherapy and immunooncology studies. In reference [29], the contribution of NF-κB in various cell types is closely examined. In the following, the effect of NF-κB proteins in tumor progression and stimulation of the immune system were discussed. Then, we discussed the relationship of this pathway with immune checkpoints to elucidate the NF-κB potential in onco-immunotherapy studies.

**Fig. 1:**
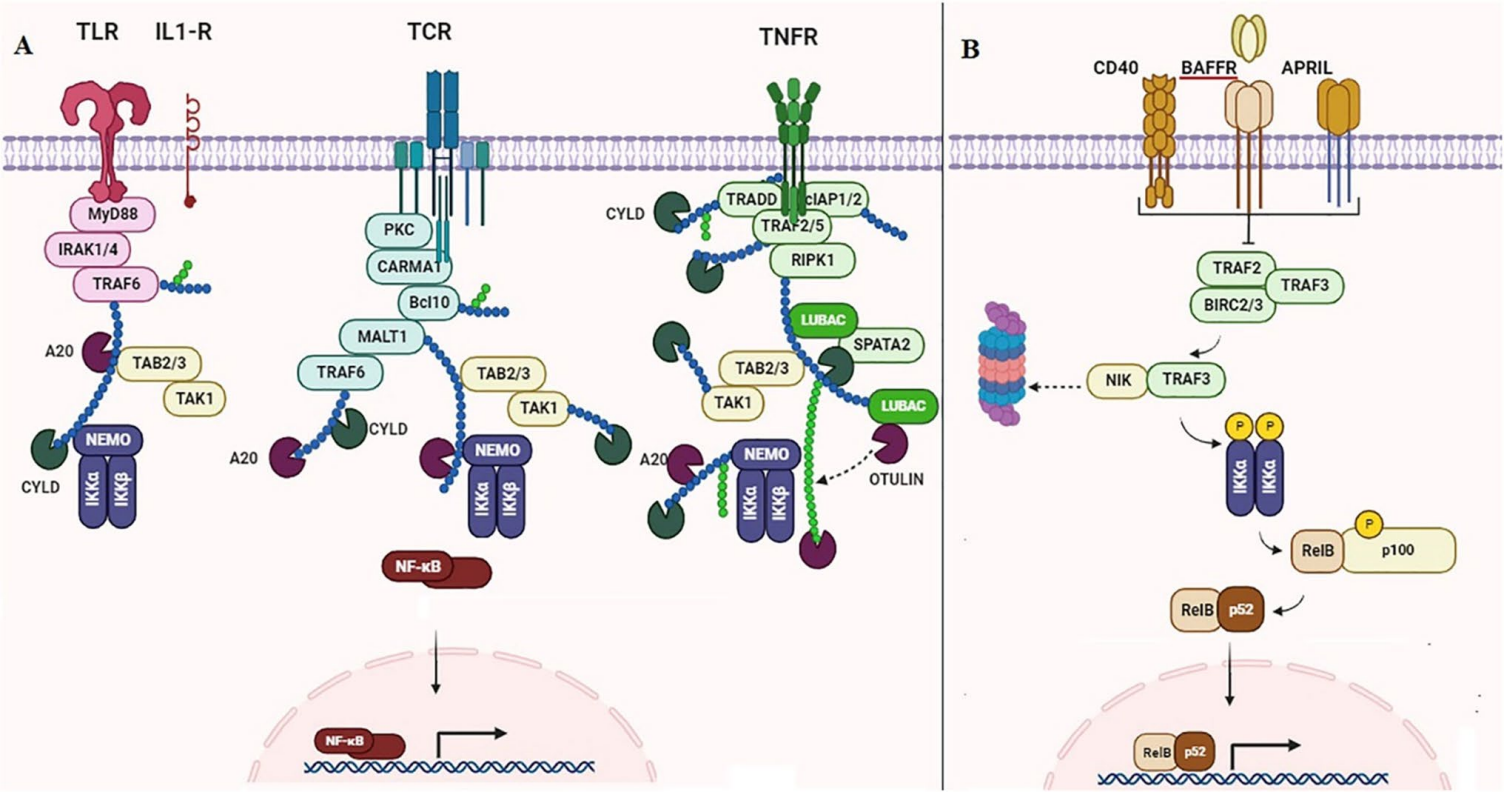
**A** canonical, and **B** non-canonical pathways of NF-κB. The canonical pathway is initiated by a variety of signals, including those conveyed by innate and adaptive immune receptors. This process entails the activation of the IKK complex through Tak1, followed by the phosphorylation of IκBα by IKK and its subsequent degradation. This leads to the rapid and temporary movement of the prototypical NF-κB heterodimer RelA/p50 into the cell nucleus. In contrast, the non-canonical NF-κB pathway relies on the processing of p100 induced by phosphorylation, which is triggered by signaling from specific members of the TNFR receptor family. This pathway is reliant on NIK and IKKα but does not involve the trimeric IKK complex. It facilitates the sustained activation of the RelB/p52 complex

Both canonical and non-canonical pathways are two different routes of NF-κB pathway. While c-Rel, RelA, and NF-κB p50 are the components of the canonical pathway, the non-canonical pathway involves P52 and RelB [30]. Receptor–antigen interactions, secretion of proinflammatory cytokines, Toll-like receptors (TLRs), and viruses are among the many inflammatory signals that can trigger the canonical pathway [31, 32]. When activated, a trimeric inhibitor of NF-κB (IB) kinase (IKK) enzyme complex phosphorylates other members of the IB family, thus activating proteins of the NF-κB complex. Following the phosphorylation of IB proteins, the rate of ubiquitination and consequent degradation by proteosomes increases, allowing the canonical NF-κB proteins to be released and translocated to the cell nucleus. Then, NF-κB triggers expression of genes that control inflammation and innate immunity in the nucleus [30, 32]. On the contrary, the non-canonical pathway is stable and slowly activated. The main inducers of the non-canonical pathway are included tumor necrosis factor receptor family ligands [30]. By stimulating the non-canonical pathway and activating IKK alpha, p100 is phosphorylated and degraded by proteasomes, leading to RelB release. In these conditions, RelB and p52 are translocated into the cell nucleus, in where expression of contributed genes in the maturation and B cells survival, and the development of secondary lymphatic organs will be regulated [30, 32]. Both canonical and non-canonical pathways of NF-κB pathway are illustrated in Fig. 1.

## The role of NF-κB in immune responses

The safety of immune responses at different stages requires meticulous control and adjustment. NF-κB is one of the essential factors regulating these responses. In addition, inflammation inevitably occurs during an immune response. The complex interactions between the innate and adaptive arms of the immune system require a precise balance [25]. The innate immune system, consisting mainly of neutrophils, macrophages and dendritic cells (DCs), acts as the first line of defense in body. Macrophages and DCs recognize foreign agents, including bacteria and viruses, and sometimes cancer antigens, through pathogen-associated molecular patterns (PAMPs) [33]. Members of the TLR family, such as TLR4, are involved in the stimulation of the NF-κB protein in response to the recognition of bacterial lipopolysaccharide [34]. The NF-κB function for the innate immune response involves the activation and translocation of NF-κB dimers into the cell nucleus. Classical dimers of NF-κB, mainly p50-RelA, increase transcription of genes encoding adhesion molecules, cytokines, and various chemokines. Furthermore, NF-κB is potent to increase enzymes' expression producing secondary inflammatory mediators and also apoptosis inhibitors [35].

Myeloid cells are essential for the immune response. TLRs and consequently, NF-κB family members are expressed in myeloid cells in high levels. Several functional abnormalities have been discovered in macrophage populations isolated from RelB^−/−^ or c-Rel^−/−^ mice. TNF-α production is impaired in RelB^−/−^ macrophages, although they may generate normal amounts of interleukin (IL)-6, IL-10, and IL-12, while overproducing IL-1β [36]. Macrophages from c-Rel^−/−^ mice generate more granulocyte–macrophage colony-stimulating factor (GM-CSF) and IL-6, but not as much TNF-α as those from wild-type mice [37].

Another function attributed to NF-κB is natural killer (NK) cells activation. For example, c-Rel can induce IL-12 producing which is released by macrophages through upregulation of p40 subunit [38]. IL-12 is essential for activating NK cells, and increases their cytolytic function and IFN-γ production [39]. Another function of NF-κBs, is their role in inhibiting LPS-induced apoptosis in macrophages [40]. Macrophages lacking IKκB, the activator of NF-κB, become highly sensitive to LPS-induced apoptosis, and are virtually ineffective [40]. Evidence has also revealed that components of the NF-κB complexes can maintain the survival of immune cells, B cells, macrophages, and T cells during bacterial attack or acute inflammation, by inhibiting apoptosis in these cells via different pathways.

## The role of NF-κB in cancer progression

Identification and clarification of the involvement of the NF-B pathway in cancer was postponed due to the rarity of genetic mutations in this pathway [41]. However, over 2 last decades, there have been numerous investigations reporting the increased NF-κB activity in a wide range of carcinomas, where it acts as an oncogene [29]. NF-κB pathway upregulation promotes many cancer-related processes, including cell death inhibition, survival enhancement, vascularization, and cell invasion [42]. Furthermore, multiple pieces of evidence have directly connected NF-B activation to the evolution of inflammatory tumors, demonstrating NF-κB potential as a viable therapeutic target in malignancy. In a pioneering study, Greten et al. [43] used a colitis-associated model of cancer in mice. They selectively inhibited IKKβ in enterocytes, which subsequently inactivated the NF-κB pathway [43]. Although these changes did not affect tumor size or mutation rates, they did reduce cancer incidence in mice by up to 80%. These results suggested that IKKβ was necessary for the primary tumor progression, but did not interfere with the onset. In these animals, the researchers blocked IKK in myeloid cells. They assumed that macrophages in the lamina propria were initially responsible for the inflammation, underscoring the importance of immunology in tumorigenesis. This therapy reduced tumor occurrence by fifty percent and significantly reduced tumor size, demonstrating that classic NF-κB activation in the was required for tumor development [43]. According to the Greten et al. study, inflammatory signaling is required in neoplastic cells, as well as in macrophages and stromal leukocytes in the cancerous microenvironment to tumor survival promotion and enhanced progression [43].

Several studies have concentrated on the role of NF-κB in regulating the expression of genes involved in cell cycle progression and escape from apoptosis. The action of NF-κB on the inhibition of apoptosis is accomplished by several mechanisms. These include, degradation or inhibition of caspases [44, 45], induction of inhibitor of apoptosis (IAP) [46, 47], BCL2 [48] production of antioxidant molecules to neutralize the effect of radical oxygen species (ROS) [49], and inhibition or reduced expression of death receptors [45]. Furthermore, NF-κB boosts the cell cycle by increasing the expression of c-myc and cyclins D3, D2, D1, and E. NF-κB transcription factors stimulates tumor associated macrophages (TAMs) in the stroma around a tumor to produce inflammatory cytokines, which decrease the maturation of DCs and inhibit the acquired immune response required for rejection of tumors [50]. Moreover, the activity of NF-κB in cancer cells promotes angiogenic pathways by increasing the expression of CXCR4 (C-X-C motif chemokine receptor 4) and CXCL8 [51]. Both CXCR4 and CXCL8 can upregulate the transcription of VEGFs [52]. During tumor invasion and metastasis, cancer cells must destroy surrounding extracellular matrix by matrix metalloproteinase and heparinase to open up space for cellular invasion and angiogenesis [53]. During this process, NF-κB promotes the expression of these enzymes and thus helps tumor invasion and migration [54]. Furthermore, the transcription of adhesion molecule like ICAM-1, in cancer cells is also induced by NF-κB, thus facilitating the migration of malignant cells to distant metastatic sites [55].

Many aspects of NF-κB pathways activation in cancer cells, the microenvironment of solid tumors, and in hematopoietic malignancies have been elucidated [56]. Besides the impact of NF-κB on gene transcription in immune cells, it directly affects cancer cells. It is involved in modulating cell cycle genes, invasion-related proteases, and apoptosis inhibitors [57, 58]. The dual contribution of NF-κB in both tumor and immune cells paradoxically results in either inhibition or progression of tumors [57, 59]. The micro-environmental conditions in association with tumor-infiltrating immune cells determine NF-κB role in the antitumor immune response [57, 60]. On one hand, infiltrating immune cells produce NF-κB-inducing cytokines. Alternatively, NF-κB can regulate genes contributed in cell survival, growth, division, metastasis, and angiogenesis [60–62]. In addition, NF-κB-induced chemokines and cytokines can cause the employment of more immunological or inflammatory cells. It also creates a positive feed-forward loop for maintain of inflammation associated with tumors [63, 64]. Moreover, development of T-regulatory (Treg) cells is a crucial task of NF-κB [65–67]. Treg cells play an immunosuppressive role in maintaining self-tolerance and immune homeostasis. New studies have shown that expression of NF-κB proteins is often correlated with the suppression of immune responses fighting against tumors. Regulatory cells, like Treg cells (which are activated by NF-κB), can inhibit the antitumor immune response if they are concentrated in the tumor [68, 69]. As mentioned, cancer cells adopt several mechanisms to maintain survival, escape from the immune system, and suppress effector cells that would otherwise kill them within the tumor microenvironment. Immune checkpoints are important factors involved in suppressing and controlling the immune system. The most studied immune checkpoints are, PD-1 and protein associated with lymphocyte T4 (CTLA-4), which are expressed on the surface of activated T cells. Binding of these receptors to their ligands (PD-L1 for PD-1, and B7 for CTLA-4) on the cell surface, inhibits T cell activity, maintains immune homeostasis, and inhibits autoimmune responses [70, 71]. In response to inflammatory signals in the tumor microenvironment, the expression of PDL-1 in tumor cells and PD-1 in immune cells is significantly increased. Under these conditions, the immune system is suppressed, and the cancer cells continue to multiply. In addition, PD-L1 upregulation is an identification marker for weak prognosis in cancer patients [72]. The CD28 stimulatory receptor interacts with the ligands CTLA-4, B7-1 (CD80), and B7-2 (CD86), resulting in the activation of T-cells. These signals activate NF-κB, which then activates T cells and ensures their survival [73]. Paradoxically protein associated with lymphocyte T4 is an alternative receptor in CD28 cells and acts as a checkpoint to suppress activation of T cell through NF-κB activity. This reduces the activity of T cells and yields an ineffective immune response against cancer cells [74]. The crosstalk between the NF-κB pathway and various immune checkpoints is described later.

The influence of NF-κB pathway activity has been observed in various cancers, such as ovarian and gastric cancer. Based on this evidence, a poor prognosis involving tumor invasion and distant metastasis has been associated with increased NF-κB activity [75]. As mentioned, mutations in the NF-κB pathway are rare, and have not been reported, except in some infection-related cancers and liquid malignancies. However, mutations upstream of NF-κB, or infection by viruses can disrupt the activity of this pathway and ultimately contribute to tumor progression [76].

## Interactions of the NF-κB pathway and immune checkpoints

The concept of immune checkpoint inhibitors (ICIs) was designed to block immune checkpoints, binding to their ligands using monoclonal antibodies (mAbs). By the blockade of ICs the T cells remain activated, and can target and destroy tumor cells in the microenvironment by secreting cytotoxic granules and effector cytokines [77–80]. Table 1 lists the known ICs and their relevant clinical trials.

**Table 1:** Immune checkpoints properties and relevant clinical trial studies in cancer research

Immune checkpoint (receptor)	Synonyms	Ligand	Expressing cells	Drugs/clinical trials	Refs.
PD-1	CD279	PD-L1	Activated T cells, B cells, NK cells, Macrophages, DCs, Monocytes, Treg cells, Langerhans cells, Tumor-specific T cells	JQ1, Atezolizumab, Nivolumab Pembrolizumab, Avelumab	[81]
CTLA-4	CD152	B7-1 (CD80) B7-2 (CD86)	CD4,CD8, Treg	Ipilimumab (IgG1), Tremelimumab (IgG2)	[82]
LAG-3	CD223	MHC class II, FGL-1, lymph node sinusoidal endothelial cell C-type lectin (LSECtin), a-synuclein fibrils, (Gal-3),	Tregs, T cells, γδT cells, invariant natural killer T (iNKT) cells, B cells, mucosal cells, associated invariant T (MAIT) cells, plasmacytoid dendritic cells (pDCs), NK cells, and neurons	Symo022/Phase I BMS-986016 (Relatimab) LAG525/ Phase 2 REGN3767 (Anti-LAG-3 mAb)/ Phase I HLX26/Phase I	NCT03489369 NCTT01968109 NCT03365791 NCT03005782 NCT05078593
TIM-3 (21kda)	HAVCR2	High mobility group protein B1 (HMGB1), phosphatidylserine, Gal9, CEACAM1	Treg cells, myeloid cells, mast cells, (IFNγ)-producing CD4 + T cells, CD8 + T cells, NK cells	MGB453 (Novartis Pharmaceuticals)/I Sym023 (Symphogen)/I ICAGN02390 (Incyte)/I	MGB453 (Novartis Pharmaceuticals) NCT03489343/NCT03652077
TIGIT	WUCAM, Vstm3, VSIG9	CD112 (Nectin-2/ PVRL2), CD155 (PVR/NECL-5),	Natural killer cells, activated CD8 + T, follicular Th cells, CD4 + T cells, Tregs,	BMS-986207/ Phase I/II Phase I/II BGB A1217/Phase I/II Ociperlimab/ Phase II	NCT05005273 NCT04570839 NCT04150965 NCT05267054 NCT05019677
ICOS	(CD278)	ICOS-L	Effector T cells, regulatory T cells	JTX-2011, GSK3359609/Phase 1/2 Feladilimab (GSK3359609): Phase 2/3 Feladilimab/ Phase 2 lung cancer study	NCT02904226 NCT04128696 NCT03739710

Among the various types of ICs, PD-1 and CTLA-4 are the most well-known, and their inhibitory antibody drugs are used clinically. Because the expression of CTLA-4 and PD-L1 are controlled differently throughout an ongoing immunological response, their ability to suppress immune responses is extremely variable. Despite many advances that have been made in ICIs, some challenges still limit their widespread use. For example, many patients do not respond to treatment with these drugs, which means they are practically ineffective. In addition, in many patients, ICIs have been associated with severe side effects [83]. The factors that regulate the transcription and advert of ICs on surface of both non-immune and immune cells are complex. In order to achieve the best efficiency and overcome the limitations of ICIs, a precise understanding of their mechanism of action and their interaction with cellular signaling pathways is necessary [84, 85]. Recent CRISPR/Cas9 investigations have revealed that the NF-κB pathway is implicated in gene expression related in immune evasion [86, 87].

Furthermore, several investigations have demonstrated that the PD-L1 gene promoter sequence contains multiple NF-κB-binding sites. This explains why this essential pathway can regulate PD-L1 expression [88, 89]. Some studies regarding the interactions between the NF-κB signaling pathway and immune checkpoints are discussed below.

### Interaction of PD-1/PD-L1 pathway with NF-κB

**Fig. 2:**
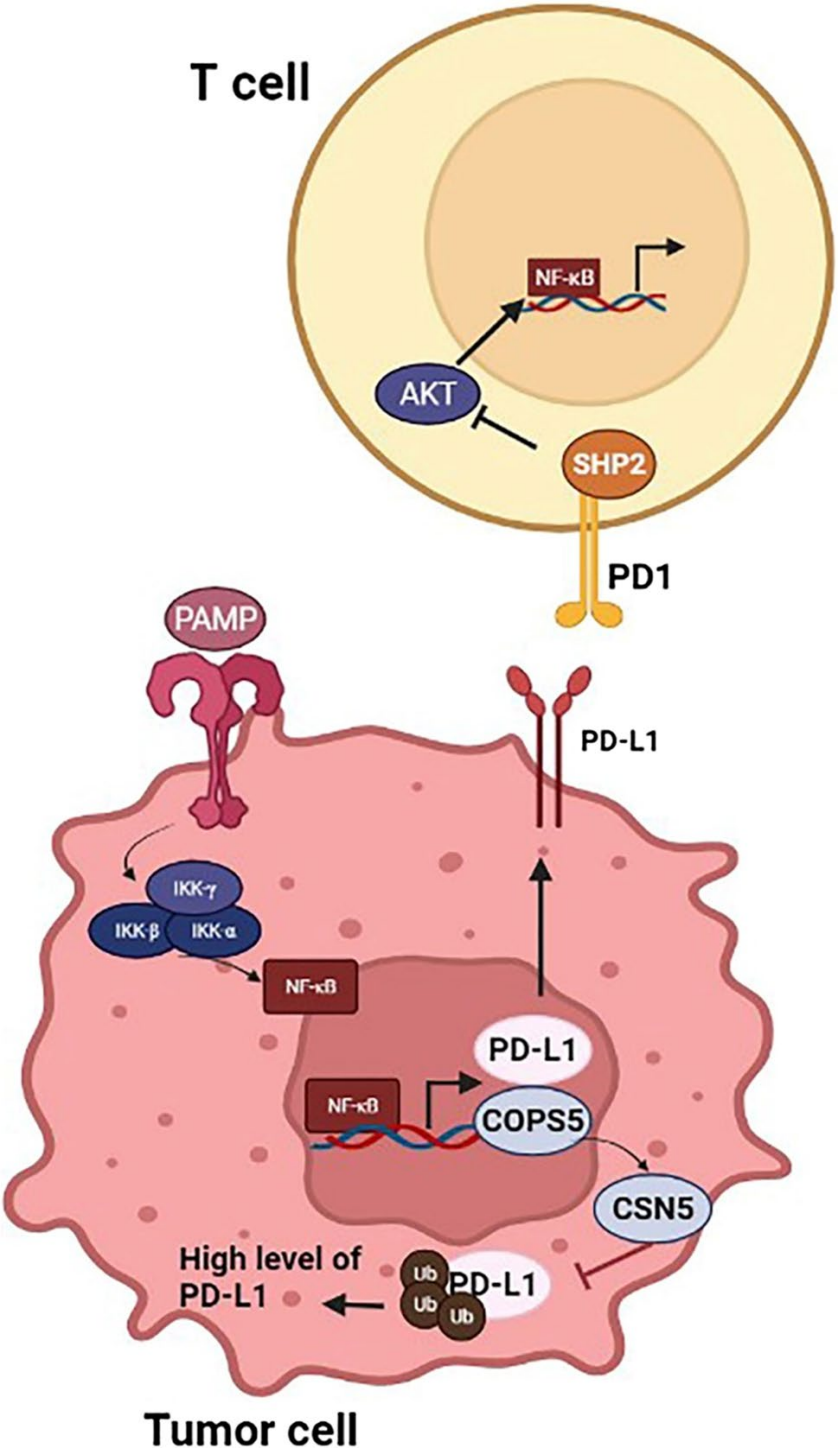
PD-1/PD-L1 signaling pathways relation with and NF-KB in immune responses

Numerous mechanisms have been described for NF-κB regulation of PD-L1 expression, both transcriptionally and post-transcriptionally [88, 90, 91]. In addition, NF-κB can increase the expression of PD-L1 by increasing its protein stability [68]. Signaling pathways dependent on TLRs play an essential role in increasing PD-L1 expression, and the escape of cancer cells from the immune response. Activation of TLR pathways and PAMPs (pathogen-associated molecular patterns) facilitates the translocation of various nuclear transcription factors such as NF-κB in cell nucleus. In addition, NF-κB binding to the promoter sequence of PDL-1 increases its expression during the transcription and translation steps [83]. The activity of TLR signaling in gastric and bladder cancer has been reported [91]. For instance, Li and colleagues [92] indicated that the recognition of lipopolysaccharide as a PAMP by TLR4, increased NF-κB activity and ultimately increased PD-L1 activity in *Helicobacter pylori*-induced gastric cancer. They also reported that NF-κB regulated gene transcription of PD-L1 by binding to the promoter via p65, leads to upregulation of PD-L1 [92]. Other mechanisms include the promotion of NF-κB nuclear translocation by interferon-gamma (INF-γ), which ultimately led to increased PD-L1 transcription. For instance, Gowrishankar and coworkers [93] reported that the upregulation of PD-L1 in human melanoma cells by INF-γ depended on NF-κB [93–95]. They showed that PD-L1 expression could be inhibited either by pharmacological methods or by genetic silencing of NF-κB using siRNA [93]. Previous research has demonstrated that INF-γ could trigger expression of the PD-L1 gene via STAT3 transcription factors [96, 97]. The involvement of STAT3 in increasing PD-L1 expression resulted from cross-talk between NF-κB and STAT3 [85]. However, after inhibiting STAT3, Gowrishankar and colleagues showed that expression of PD-L1 did not change conspicuously and, therefore, suggested that the ability of NF-κB to regulate PD-L1 was independent of STAT3 [93] (Fig. 2).

As mentioned above, the NF-κB pathway is closely associated with inflammation, and its increased activity has been observed in most inflammatory cancers [98]. The combined activity of the EBV-associated latent membrane protein 1 (LMP1) and IFN could further boost PD-L1 expression in Epstein–Barr virus (EBV)-positive nasopharyngeal carcinoma cells [99]. LMP1 is an activator the NF-κB pathway, and increases the expression of PD-L1 by activating AP-1, STAT3, and NF-κB [100]. Fang and colleagues showed that the inhibition of NF-κB could down-regulate PD-L1 expression via LMP1 in nasopharyngeal carcinoma cells [99]. Li et al. found that IFN-γ secretion was linked to NF-κB activation. However, their findings pointed to a significant role for the JAK/STAT1 pathway in the expression of PD-L1 in HCC cells. In addition, they reported a synergistic increase in transcription of PD-L1 via a combined activity of TNF-α and IFN-γ. TNF-α is thought to upregulate IFN-γ receptor expression through NF-κB pathway, leads to increased signaling of IFN-γ in hepatocellular cancer cells, thus driving cancer progression [101]. Wang et al. examined the effects of activating the NF-κB pathway on PD-L1 expression in clones of prostate cancer cells [102]. They found that TNF-α and IL-17 could synergistically upregulate PD-L1 via NF-κB activation [102]. The PD-L1 transcription could be epigenetically adjusted by methylation of DNA sequence plus NF-κB through EMT in NSCLC, according to one report [103]. DNA methylation and NF-κB signaling, according to this theory, acted together to affect the expression of PD-L1. For enhancement in the demethylated promoter of *PD-L1* gene, the treatment required both TNF-dependent activation of the NF-κB pathway and demethylation of the PD-L1 promoter, leading to more NF-κB binding to PD-L1 promoter [103].

### Tumor suppressor genes or oncogenes in NF-κB pathway can regulate PD-L1 expression

Many factors affect the binding of NF-κB to specific promoter elements. For example, a member of the IKB family, Bcl3 a known oncogene, is located mainly inside the nucleus with an activation domain. Activation of Bcl3 depends on a combination of transcriptional regulators and NF-κB subunits binding to the elements. The NF-κB-mediated trans-promoter responder can activate or suppress specific genes [104–107]. An association between Bcl3, NF-κB pathway, and PDL1 expression has recently been reported in ovarian cancer cells [104]. Bcl3 upregulated PD-L1 expression in ovarian cancer cells mediated by IFN-γ [89, 108]. Various molecular results indicated that the transcriptional co-activator p300 connects to the promoter sequence of PD-L1 gene. IFN-γ pathway activation upregulated Bcl3, leading to more acetylation of p65, and its binding to the promoter of PD-L1 independent of p300. Under these conditions, the transcription and expression of PD-L1 are significantly increased [89]. In cells of triple-negative breast cancer, the expression of PDL1 was dependent on an oncogene named mucin1 (MUC1) [109]. The cytoplasmic domain of MUC1, a transmembrane glycoprotein, is responsible for integrating pathways implicated in the formation and progression of various malignancies [110]. It has recently been found that MUC1 can activate NF-κB expression in many cancers. In addition, MUC1 is involved in NF-κB translocation to the nucleus. On the other hand, MUC1 can bind directly to NF-κB and govern the transcription of its target genes [111–113]. Maeda and coworkers indicated that transcription of PD-L1 was increased by MUC1 by activating NF-κB and Myc in triple-negative breast cancer [109]. Recently, the role of retinoblastoma (RB) a tumor suppressor, in the regulation of NF-κB expression and subsequently PD-L1 has been reported [114]. When RB is phosphorylated at threonine 252 and serine 249, it reacts faster with p65, and prevents connecting to the promoter sequence of PD-L1 agene. Mutations in RB, or inhibition of its phosphorylation at the mentioned sites can increase activation of NF-κB and lead to the PD-L1 up-regulation. For some patients, radiotherapy can increase PD-L1 expression [115]. It was proposed that radiation could reduce phosphorylation at the mentioned sites, eventually leading to NF-ΚB activation, and more upregulation of PD-L1 [114, 116–118]. Mutations in the epidermal growth factor receptor (EGFR) are often correlated with cancer therapy tolerance in lung cancer. Recently, a strong association has been reported between higher expression of PD-L1 and EGFR mutations in NSCLC [119, 120]. This upregulation was associated with the enhancement of phospho-IKBα and HIF-α (hypoxia-induced factor 1) [121, 122]. Therefore, it was suggested that there was a close relationship between NF-κB and IFN-α in PD-L1 regulation [121]. Indeed, direct connecting of NF-κB to the promoter sequence of HIF-1 gene, as well as HIF-1-dependent NF-κB activity, have been observed [121, 123, 124]. Lin and coworkers [125] proposed that NF-κB pathway, EGFR, and PD-L1 transcription were all linked. These researchers discovered an increase in the expression of NF-κB in EGFR-mutant cells compared to EGFR-wild type cells, as well as a link between activation of EGFR and high level of PD-L1 transcription. Furthermore, EGFR-tyrosine kinase inhibitors (EGFR-TKI) could also inhibit NF-κB, and downregulate PD-L1 in non-small lung cancer EGFR mutant cells. However, more research is needed to better understand the interactions of NF-κB pathway and EGFR in the control of PD-L1 transcription [125]. PD-L1 deubiquitinization by fifth element of the Cop9 signalosome protein (CSN5) maintains stability of PD-L1 [126]. Lim et al. showed that NF-κB p65 was activated by TNF-α and bound to the promoter of the Cop55, thereby increasing the expression of CSN5. Direct binding of CSN5 to PD-L1 can prevent its ubiquitination and proteasomal degradation. In addition, in nasopharyngeal carcinoma, p65 and STAT3 both bind to the promoter sequence of CSN5 gene, ultimately increasing the expression and stability of PD-L1 [126]. Macrophages have been shown to induce the formation of the NF-κB p65/STAT3 complex in colorectal cancer via the CC-chemokine ligand 5 [127, 128]. Although studies on the role of NF-κB in post-translational regulation of PD-L1 are limited, it has been clearly demonstrated that NF-κB can regulate PD-L1 expression at different levels.

## Correlation between CTLA-4 and the NF-κB pathway

CTLA4 was first identified as an inhibitory signal responsible for terminating immune responses. CTLA4 is expressed as a transmembrane protein in active effector T (Teff) cells. It ultimately suppresses T cell-dependent immune responses, including T cell proliferation and the secretion of inflammatory cytokines, such as INF-γ and IL-2 [129]. The mechanisms of action of CTLA4 in suppressing the immune response are not yet completely understood; however, several mechanisms have been proposed. For example, CTLA-4 could compete with CD28 + for binding to their common co-stimulation ligand B7, expressed on surface of APCs. CTLA-4 in addition, can suppress division of immune cells such as T cells by its cytoplasmic domain and its effect on signaling molecules [130]. In recent years, the blockade of CTLA-4 has been investigated as a cancer immunotherapy approach, and has been a target for immune checkpoint inhibitors. Anti-CTLA4 monoclonal antibodies, including tremelimumab and ipilimumab, have been used alone or in combination with other cancer therapies like radiotherapy, anti-cancer vaccines [6], or chemotherapy [131]. In addition, anti-CTLA4 drugs have been studied in multiple preclinical studies and clinical trials, combined with anti-PDL1 or anti-OX40 antibodies for various cancers, including NSCLC [132], prostate cancer [132], mesothelioma [133], breast cancer [132], HCC [134], and pancreatic cancer [135]. Evidence suggests that CTLA4 expression is mainly shown in induced T cells, especially memory and Treg cells [136]. Foxp3 + Treg cells also account for much of the expression of CTLA4 and its effect on immunosuppression [137, 138]. Foxp3 (forkhead box P3) is a transcription factor essential for the proper function and development of Treg cells [139]. According to recent studies, Foxp3 is responsible for the expression and stability of CTLA4 [138]. Treg cells inhibit the activity of Teff cells and suppress cellular immune responses [137].

Numerous investigations demonstrated that proteins of NF-κB, particularly C-Rel, is essential for Foxp3 transcription and maturation of Treg cells in the thymus [140–142]. NF-κB complexes containing c-Rel subunits essentially contribute in the immune response and lymphoid development. Considering the dual role of NF-κB in inducing an antitumor immune response against cancerous cells, and inducing the expression of immune checkpoints in Treg cells, employment of NF-κB suppressors for cancer immunotherapy is challenging [143, 144]. It has been suggested that the use of inhibitors that target only the NF-κB c-Rel subunit could open a new avenue in cancer treatment [145]. Grinberg et al. [68] showed that explicit targeting of c-Rel prevented tumor progression, and protected activated Treg cells present in the tumor site. Using an engineered mouse model lacking the c-Rel subunit, they showed that melanoma progression was significantly inhibited in these mice. Furthermore, chemical inhibition of c-Rel activity slowed the progression of melanoma by reducing immunosuppression which are mediated by Treg cells, and amplified the impacts of anti-PD-1 treatment. Inhibition of NF-κB c-Rel could therefore be an approach to improve checkpoint-targeted immunotherapy, according to Grinberg et al. Although this study focused on the association between PD-1 and NF-κB c-Rel, its role in the development of Treg cells by upregulation of Foxp3 expression, as well as the interaction of Foxp3 with CTLA4 might be the focus of future research [145].

**Fig. 3:**
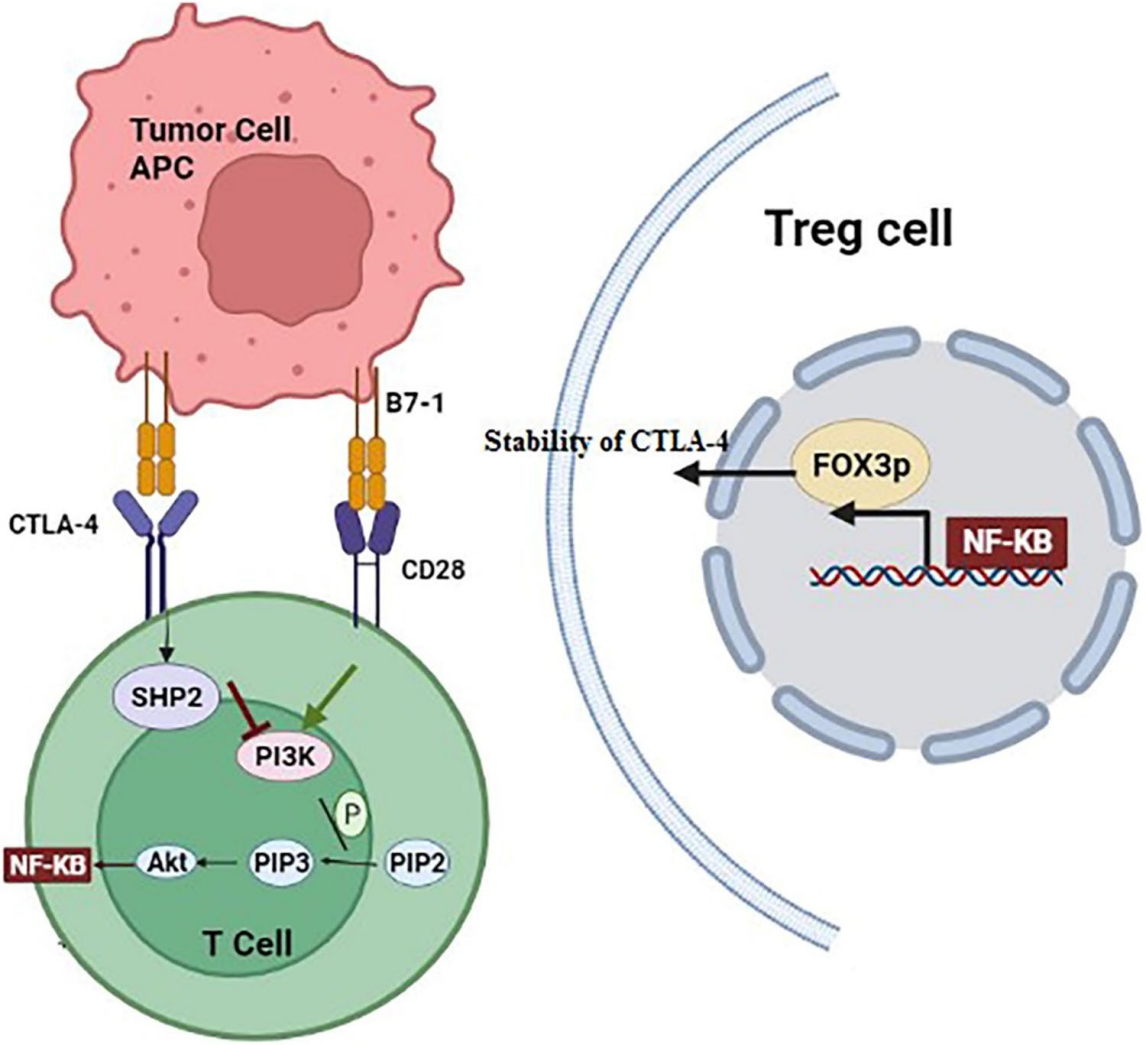
Relationship between CD28 and CTLA-4 pathways and interactions with NF-κB

Nevertheless, so far recent results of studies on NF-κB and CTLA4 are rather complex. On one hand, NF-κB can contribute in the expression of CTLA4 and the formation and maintenance of Treg cells, which ultimately results in the silencing of the immune response against tumors. On the other hand, according to current research, CTLA4 can inhibit cellular pathways, including PI3K/AKT, cyclin-dependent kinases (cdk4/cdck6), cyclin D3, and NF-κB, which all induce Teff cells. Under these conditions, T cell division and activity are effectively inhibited, and the immune response against the tumor is suppressed [146, 147]. However, the central relationship between CTLA4 and NF-κB has been studied through the CTLA4/CD28 signaling pathway. Activation of T cells occurs through two different pathways. One is the binding of antigens presented by APCs with the cognate TCRs on T cells, while the other is related to the CD28 costimulatory molecule. After CD28 binds to the B7 molecule in APCs, a TCR activation signal is induced, which leads to clonal proliferation of T cells and the initiation of effector cellular immune responses [148, 149]. As mentioned, CTLA-4 competitively binds to B7, thus preventing the CD28 signaling and modulating the immune response. The CD28 signaling eventually activates PI3K/Akt signaling. This cellular signaling pathway activates cell proliferation and inhibits apoptosis, via BCL-XL, mTOR, and NF-κB intermediates (Fig. 3). In contrast, studies have shown that CTLA4 signaling activity in Teff cells activates SHP2 protein, which acts as an inhibitor of the PI3K/Akt pathway, and its downstream elements, such as mTOR and NF-κB [150]. Therefore, in Teff cells, apoptosis is increased, cell division is decreased, and cellular immunity is suppressed. As mentioned, anti-CTLA4 antibodies are currently used clinically in combination with other treatments [151]. For example, in combination with nivolumab (an anti-PD-L1 antibody), ipilimumab was more effective in advanced melanoma cancer than when used alone [152].

Furthermore, in recent years, anti-CDLA4 antibodies have been combined with other agents that target NF-κB, and with denosumab, an antibody against RANKL (receptor activator of nuclear factor-kappa-Β ligand) [152], as described below.

### LAG3 and NF-κB pathway in immune response

Lymphocyte activation gene 3 (LAG 3) is another critical immune checkpoint, and a member of the immunoglobulin superfamily, which has been linked to cancer, autoimmune diseases, and infections [153]. LAG3 is an ancestral homolog of CD4 and is also connected to MHC Class II. In addition, the lectin galectin-3 (Gal-3), α-synuclein fibrils, and FGL-1 are also LAG3 ligands. Like other immune checkpoints, LAG 3 inhibits the immunological response by reducing the production of cytokines and granzyme in T cells, and promoting their differentiation into Treg cells [154, 155]. After PD-1 and CTLA-4, LAG3 is the most important immune checkpoint to be investigated in immunotherapy studies, and the LAG3 blockade approach is in the clinical trial phase [156]. Despite some promising studies conducted in recent years on the role of LAG3 in cancer, especially melanoma, there are still many unanswered questions, most of which are related to the action mechanism of LAG3. Therefore, it is necessary to understand the relationship of LAG3 with cancer-related signaling pathways and immune pathways, including NF-κB. High level expression of LAG3 is detected in many immune cells such as, T, Treg, γδT, B cells, plasmacytoid DCs (pDCs), and natural killer (NK) cells [157–159]. Cytokines, especially interleukins 2, 7, 12, 15, and 27, as well as TCR, can increase LAG3 expression [160]. In addition, other transcription factors, including NF-AT and TOX, can regulate the expression of LAG3, and induce the transcription of other immune checkpoints [161, 162]. However, the exact relationship between LAG3 and NF-κB has not yet been fully elucidated, although there does seem to be a complex relationship. The inhibitory function of immune checkpoints involves the suppression of T cells through their cytoplasmic domains with inhibitory roles like ITIM and ITSM. Both of these domains bind to phosphatases with Src homology 2 (SH2)-binding domains, in the immunological synapse to counteract TCR activation signals relayed by kinases [163–165].

Because LAG3 has no inhibitory domain, it is unique among immune checkpoints. In contrast, the cytoplasmic domains of LAG3 contain three conserved regions in both humans and mice (EX/EP motifs, PRFSALE, and KIEELE motifs). Although the exact function of these motifs in the inhibitor activity of LAG3 has not yet been elucidated, one protein has recently been identified called LAG3 associated protein (LAP) that binds to the EP motif, and could explain the mechanism of LAG3 affecting NF-κB. It is proposed that EP motif can cluster LAG3 into lipid rafts. In addition, this motif is contributed in translocation of LAG3 from microtubule organizing center to surface of cells through packing in endosomes. Nevertheless, EP motif deletion had no effect on expression of LAG3 on the surface of immune cells, nor did an inhibitor of tubulin polymerization, implying that LAG3 is trafficked by other routes [165]. The > 99% similarity between LAP and the C-terminus of CENPJ has led to speculation about the role of the LAP, suggesting it may be involved in the organization of centrosomes and microtubules during cell division, well as increasing STAT5 and NF-κB signaling [166, 167]. CENPJ is a member of the centromere protein family, and plays a structural role in maintaining the integrity of centrosomes and in microtubule disassembly. Furthermore, CENPJ acts as a transcriptional activator in the STAT3 pathway. In addition, CENPJ is an NF-κB-mediated transcriptional co-activator via its interaction with the P300KREB binding protein [168]. Because in response to IL-7 or after peptide stimulation, LAG3 has potential to decrease phosphorylation of STAT5 and Akt, one unconfirmed hypothesis is that the function of the EP motif of LAG3 is to prevent LAP from acting as a coactivator for STAT5 and NF-κB [169]. This may explain why LAG3 can promote cell cycle arrest [170]. LAG3 and NF-κB could potentially bind to each other via Gal-3, known to be a LAG3 ligand. Gal-3 is a type of lectin that inhibits immune checkpoints by filling the binding site of LAG3 in CD8 + T cells. Gal-3 is highly expressed during inflammation and strongly binds to macrophages. Gal3 has many roles in cell division, apoptosis, mRNA editing, inflammation, angiogenesis, and metastasis. In addition, Gal-3 may be involved in suppressing T cell lysis [171]. According to a study by Kouo et al., Gal-3 interacted with LAG3 in CD8 + T cells in pancreatic adenocarcinoma [172]. Numerous studies have investigated the role of Gal3 as a marker for the overall survival of ovarian, NSCLC, colorectal, and uveal melanoma (UM) cancer patients [173]. The results also showed that knockdown of Gal3 reduced signaling pathways in endothelial cells. According to these results, increased expression of Gal3 and LAG3 was observed in some cancers, like UM, and could be a reason for the reduced CD8 T cell activity. Besides, Gal3 is also involved in the growth of cancer stem cells. Gal3 increased signals implicated in the stemness of cancer cells, including the NF-κB pathway [174–176]. Gal3 is an essential activator of the NF-κB pathway in cancer cells. More studies are needed to show whether Gal3 can simultaneously affect LAG3 and NF-κB signaling in immune cells. Moreover, it is unclear whether the interaction of LAG3 with Gal 3 affects NF-κB expression?

## NF-κB and the T-cell immunoglobulin mucin domain 3 (Tim-3)

Tim-3 is a cell-level domain immunoglobulin found in IFN-γ-secreting CD4 + Th1 and CD8 + T1 cytotoxic cells [177]. Tim-3 is involved in both allergy and autoimmune responses [178, 179]. Tim-3 was described by Monney et al. as an inhibitory receptor for T cells, because the injection of anti-Tim3 monoclonal antibodies increased the severity of autoimmune disease, in the experimental autoimmune encephalomyelitis mouse model of autoimmunity [177]. In subsequent investigations, Tim-3 was found to be an immune checkpoint. Notably, Tim-3 is usually expressed along with other ligands for ICs such as TIGIT, PD-1, and LAG 3 [180, 181]. Expression of Tim-3 distinguishes the most dysfunctional or terminally exhausted subgroup of CD8 + T cells in models of tumors [182, 183]. Preclinical studies revealed that concomitant inhibition of Tim-3 and PD-1 pathways has a more significant effect against solid and hematologic tumors [184, 185]. Although not yet clear, the proposed mechanism of Tim-3 activity, involves HLA-B-associated transcript 3 (Bat3), and SH2 interacting with conserved tyrosines including Y256 and Y263, in Tim-3 cytoplasmic tail [186]. This immune checkpoint is involved in immune synapse by activating T cell signaling. Bat3 connects to the Tim-3 cytoplasmic tail, where it serves as the active catalytic form of the lymphocyte-specific tyrosine kinase protein [186]. Decreased expression of Bat-3 leads to a more robust transmission of inhibitory signals from Tim-3 [187]. However, it should be noted that Tim-3 signaling has only been characterized for T cells, and its effect on other cells, including dendritic cells, should be further investigated. Src homology domains (c-Src and SH2), which are signal transducers for Bruton's tyrosine kinase are activated by Tim-3 ligation in DCs, leading to inactivation of NF-κB and inhibition of DC activation [188]. However, Tim-3 function in DCs is unknown, preclinical investigations and animal model studies revealed that Tim-3 has the ability to reduce cytoplasmic TLR-induced stimulation. In result, blockade of Tim-3 improves chemotherapy results by activating DCs [189, 190]. Anti-Tim-3 antibodies caused tumor DCs to produce more CXCL9, which increased activation and lymphocyte infiltration in animal models of breast cancer [189]. Antibodies against galectin-9, were shown to enhance CXCL9 production by tumor cDC1s. In agreement, Tim-3 ligation has been demonstrated to inhibit the maturation of murine DCs by interfering with NF-κB activity [188].

Studies on the crosstalk between Tim-3 and NF-κB pathways are somewhat controversial. This may be because of the critical significance of NF-κB in inflammatory pathways and its different functions in different cells. Lin and colleagues [191] investigated the action mechanism of Tim-3 of in mice with acute pancreatitis in the early stages. As mentioned above, TLRs are the primary receptors for NF-κB pathway activation, and can act as an innate or acquired immune interface for signal transduction, pathogen detection, and immune response activation. TLR4 binds to a critical adaptor protein called MyD88, which activates NF-κB and causes a cascade of inflammatory cytokines to be produced [192]. The binding of TLR4 to the PAMP ligand activates the NF-κB pathway and thus increases inflammatory signals [193]. Lin and coworkers indicated a decrease in the expression of Tim-3 that can result in increasing of expression of NF-κB, IL-6, and TNF-α and increased the severity of the disease [191]. Indeed, Tim-3, as a modulator of the immune response and macrophages, inhibits NF-κB expression and inflammatory signals [191]. In contrast, Lee et al. [194] reported different results. They found that Tim-3 was involved in suppressing the expression of TNF-α, INF-γ, and IL-2 in CD8 + T cells and inhibiting the nucleus trafficking of NFAT in these cells. These effects led to the induction of an exhausted phenotype in T cells, but had no effect on NF-κB expression or translocation into the nucleus.

A study by Liu et al. [195] also revealed a correlation between Tim-3 and NF-κB expression in macrophages involved in liver cirrhosis. Furthermore, they found that Tim-3 expression was reduced in cirrhosis patients than in the healthy group. The results showed that the abnormal function of macrophages in cirrhosis was due to an impaired FTX/miR-545 signaling pathway. Indeed, the long non-coding RNA lncRNAFTX increased the expression of Tim-3 by inhibiting miR-545, which in turn reduced translation of TNF-α, IL-6, and NF-κB. This study also revealed that Tim-3 had an inhibitory role in NF-κB expression during inflammatory responses. In addition, studies on the role of Tim-3 in liver damage showed an association with NF-κB. Tim-3 bound to its natural ligand, galactin 9 (Gal-9), and suppressed the activity of Th1 cells by inducing apoptosis [196]. Interestingly, some results suggest that Tim-3 expressed in innate immune cells may operate synergistically with the TLR system to enhance inflammation. Expression rate of Tim-3 is enhanced in terminally differentiated Th1 cells after activation, whereas upregulation of Gal-9 might decrease Th1 immunity and affect a variety of inflammatory conditions. As a result, Tim-3 could play opposite roles in innate and adaptive immune cells, causing divergent signals [197]. Tim-3 inhibited the TLR-dependent immune responses due to its immunosuppressive role [197]. One investigation by Zhao and colleagues [196] reported that the administration of mAbs against Tim-3 upregulated TLR4 and consequently NF-κB pathway activity in liver tissue, and led to increased inflammation. Disruption of Tim-3 signaling enhanced transcription and activation of NF-κB, and the liver tissue displayed apoptosis, hepatocyte shrinkage, and necrosis compared to controls [191].

TIM-3 cross-linking by an anti-TIM-3 antibody reduced DC activation and maturation by inhibiting the NF-κB pathway, according to Maurya et al. Following Ab-mediated cross-linking, TIM-3 was tyrosine phosphorylated, and the non-receptor tyrosine kinases, Bruton's tyrosine kinase (Btk) and c-Src were successively recruited and activated. The stimulation of Btk–c-Src signaling resulted in the production of inhibitory factors by DCs, which blocked the NF-κB pathway, preventing DC activation and maturation. TIM-3's inhibitory actions on DCs were abolished when Btk or c-Src were silenced. These findings show that Btk–c-Src signaling is critical for TIM-3-induced DC inhibition [188]. Kikushige et al. also demonstrated a relationship between Tim-3/Gal-9/NF-κB axes in human myeloid leukemia stem cells. It is approved that NF-κB is able to increase Wnt signaling, causing epithelial non-stem cells to dedifferentiate and become tumor-initiating cells. Considering this results, Kikushige’s team [198] reported that an autocrine signaling pathway of TIM-3/Gal-9 activated the canonical Wnt signaling in myeloid leukemia stem cells. They discovered that TIM-3 was a specific surface marker unique to leukemic stem cells (LSCs). They found that the TIM-3/Gal-9 pathway was a key stimulatory loop for LSCs, because AML cells produce a large amount of Gal-9 in patient serum. Both NF-κB and β-catenin pathways are activated through TIM-3 signaling activation. Human AML reconstruction was hampered by Gal-9 neutralization in immune-deficient mice in one investigation. Kikushige et al. showed that β-catenin translocation to the nucleus occurred in TIM-3 + AML cells in response to Gal-9 ligation [198]. Therefore, the results regarding the relationship between NF-κB and Tim-3 during immune responses and various cancers are contradictory [198]. Therefore, more accurate and more detailed research is necessary.

## TIGIT (T cell immunoreceptor with Ig and ITIM domains) is able to inhibit NF-κB pathway

TIGIT is expressed on the surface of T cells and NK cells. This immune checkpoint protein contains an Img variable domain, transmembrane domain, and tyrosine-based immunosuppressive inhibitory motifs [199, 200]. TIGIT, together with its two main ligands, poliovirus receptor (PVR) and nectin-2, has a potent inhibitory effect on the activation of T cells and NK cells [200, 201]. The immunoreceptor motif or ITIM plays a critical role in the strength of the inhibitory signal and controls the activity of immunological cells [201]. TIGIT has been identified as an immune checkpoint in several autoimmune diseases and cancers [202]. TIGIT binds to PVR and leads to the secretion of IL-10 in human DCs, which ultimately suppresses the activity of T cells. Concomitant blockade of both PD-1 and TIGIT intensifies the immunological response versus melanoma, so melanoma cells’ inhibition is much more effective than the use of anti-PD-1/PD-L1 alone in melanoma patients [203]. The results show that TIGIT has an innate ability to inhibit and suppress T cells [204]. TIGIT has a typical ITIM motif that mediates negative signaling by connecting to SH2 domain in protein tyrosine phosphatases including SHP1 and SHP2, or protein inositol phosphatases such as, SHIP1 and SHIP2 [205]. Moreover, TIGIT possesses an ITT-like motif in the cytoplasmic domain. These motifs provide the foundation for the cytosolic adaptation of Grb2 or phosphatidyl 3-kinase (PI3K) class IA. So far, over than 30 Grb2-related proteins have been detected in immune cells [206]. Furthermore, studies show that the ITT motif in the cytoplasmic part of IgG and IgE triggers ITAM-induced signals and leads to increased division rate of B cells. Nevertheless, the way that ITT-like motifs work is unclear in T cells and in NK cells [207].

There are only limited studies on the association between NF-κB and TIGIT pathways. After TIGIT/PVR ligation, recruitment of Grb2 and SHIP1 via the ITT-like motif inhibits the NF-κB, PI3K, and MAPK pathways, resulting in the loss of NK cell activity [208–210]. Li and colleagues [208] suggested that the binding of TIGIT to its ligand PVR, inhibited production of IFN-γ in natural killer cells. Furthermore, they reported that transgenic NK cells expressing high levels of TIGIT produced less IFN-γ during binding to PVR. In addition, in NK cells without TIGIT (knockout), the level of IFN-γ releasing is much higher. Indeed, TIGIT/PVR signaling significantly reduced the level of IFN-γ releasing by inhibiting NF-κB signaling. They discovered a new adaptor called arrestin 2, which binds to phosphorylated TIGIT and recruits SHIP1 via an ITT-like motif. SHIP1 inhibits autoubiquitination of TNF receptor-associated factor 6, which prevents activation of NF-κB and suppresses releasing of IFN-γ in natural killer cells. Although these findings could provide valuable clues about the relationship between TIGIT and NF-κB, further studies focusing on cancer are essential to find new approaches to cancer treatment [208].

## Inducible T-cell costimulator (ICOS) can be regulated by canonical NF-κB

In 1999, a 60 kDa homodimer protein was discovered on the surface of TCR-stimulated T cells, which was called inducible T-cell costimulator (ICOS). This immune checkpoint is a member of the CD28 superfamily, and has a close resemblance to CD28. However, one of the main differences between these two pathways is the lack of consistent expression of ICOS on all T cells. ICOSL is a specific ICOS ligand expressed on various APC cells, including DCs, macrophages, and even on other non-lymphoid tissue-derived cells including endothelial fibroblasts, and epithelial cells [211]. Furthermore, numerous investigations have studied the expression of ICOS in other T cell types, including Tfr (T follicular regulatory cells), Tfh (T follicular helper), Th1, Th2, Th17, Tr1 (type 1 regulatory T), Treg, and ILCs. ICOS expression on this wide range of cells indicates its vital role in immune responses [212–214]. The role of ICOS in various diseases is difficult to comprehensively explain, given the intricacy of ICOS distribution on T cell subsets and its diverse effects on each subset. Overexpression of ICOS may trigger either Th1 or Th2 immunological responses, with Treg cells serving as a critical balance in inflammatory signaling [215]. One of the prominent roles of ICOS in acting as an immune checkpoint and suppressing the immune response is its ability to regulate Treg cell functions [216]. ICOS-expressing Treg cells show increased expression of several genes in correlation with stimulation of TCR, and have a more pronounced proliferation potential. ICOS expression appears to be influenced by transcription factors contributed in the differentiation of Treg cells, and contributes to the biology of effector Treg (eTreg) cells [217]. For example, the regulation of eTreg cell activity and tissue homeostasis is controlled by Blimp1 (B lymphocyte induced maturation protein 1). Blimp1 is essential for the production of IL-10 and transcription of ICOS [218]. IRF4 (interferon regulatory factor 4) is also known as a differentiating factor for Treg cells, and the suppression of the Th2 response by Tregs [218, 219]. Studies have shown that in Treg IRF4^−/−^ cells from chimeric mice, ICOS expression is lost [218]. In addition, some studies have shown a role of NF-κB in regulating ICOS. NF-κB plays an essential role in the phenotype and function of T cells, which is mainly performed through its two canonical subunits, RelA, and c-Rel. c-Rel is critical for maturation of thymic Tregs, and the function of activated Tregs (aTregs). In Treg cells that lack c-Rel expression, the expression of correlated genes with homeostasis and aTregs’ function were significantly reduced. At the same time, relA is responsible for the maturation, maintains, and activation of eTregs [68, 145]. Tregs with inclined RelA expression were shown to have a substantial decrease in transcription of eTreg signature genes including *Tigit*, *ICOS*, and *Il10* that were similar to IRF-4-deficient Tregs. RelA, on the other hand, regulates eTreg production and function independently of IRF4. TNF receptor superfamily (TNFRSF) signaling, such as GITR or TNF signaling, has been shown to activate RelA in the absence of TCR signals, thereby controlling Treg’s activity [220]. Although studies on ICOS regulation by NF-κB are limited, this immune checkpoint appears to be strongly influenced by the NF-κB pathway.

## Anti-RANKL agents combined to ICIs

Due to the multiple mechanisms by which cancer cells can escape immune attack, there has been a strong focus on combining immunotherapy with immunomodulatory drugs to increase the susceptibility of cancer cells to immunotherapeutic agents [221]. Recent studies have shown that RANK/RANKL inhibition can be a practical approach to increase the effectiveness of immunotherapy. RANKL is a cytokine of the TNF-α superfamily, and is commonly expressed in dendritic and T cells. The RANK–RANKL interaction caused receptor trimerization and to recruitment of TRAF adaptor proteins. This ultimately activates downstream pathways, including Act/PKB, ERK, MAP kinase cascade, and NF-κB [222]. RANK-expressing cells are found in large numbers in the tumor niche. In addition, it is indicated that RANK contributed essentially in both innate and adaptive immune responses. Treg cell production and cytokine secretion are the most critical consequences of activation of RANK/RANKL signaling [223]. Smyth et al. [224] used denosumab, an anti-RANKL antibody combined with ipilimumab (anti-CTLA4 antibody) to treat a metastatic melanoma patient with highly invasive bone metastases. This treatment regimen showed a relatively effective response and kept the patient alive for more than two months. These researchers demonstrated that anti-RANKL and anti-CTLA-4 mAbs have modest antimetastatic activity when applied alone in a preclinical mouse model of B16F10 melanoma with experimental metastases, but the metastasis suppression was significantly improved when these drugs were used in combination at the time of intravenous melanoma inoculation [224]. Recent findings approved that the efficiency of anti-CTLA4 and anti-RANKL combination therapy depended on lymphocyte functions. In mouse models without lymphocytes or without DCs, this combination was entirely ineffective. According to a recent retrospective analysis, the addition of denosumab to immune checkpoint inhibitors in melanoma patients could be promising for better treatment outcomes [225]. In a study by Ahren et al. [226], the mechanism behind these results was investigated to some extent. They evaluated the effectiveness of the combination of CTLA4 and RANKL inhibitors by analyzing tumor growth, tumor-infiltrating lymphocytes, and metastasis in mouse models. They used various neutralizing antibodies and mouse models with different knockout genes. Finally, it was shown that blocking RANKL could increase the effectiveness of anti-CTLA4 antibodies in solid malignancies, as well as investigational metastases [226].

Anti-CTLA-4 mAbs that acquired from mouse IgG2a isotype could deplete Tregs and exhibited the best combinatorial efficacy with anti-RANKL. The existence of activating Fc receptors and lymphocytes (especially NKs and CD8 + cells) was required for the best effects, but the use of anti-RANKL alone had no effect. In solid tumors, the injection of anti-RANKL and anti-CTLA 4, produced a higher infiltration of T cells into tumors, and enhanced practical T functionality. Denosumab combined with ICIs is going to be tested in metastatic or unresectable melanoma or renal cell carcinoma patients in current launched clinical trials including CHARLI (NCT03161756) and KEYPAD (NCT03280667). The PERIDENO (NCT03532087) trial in postmenopausal patients with HER2^−^ breast cancer combined to AC-T chemotherapy in association with denosumab monotherapy in cervical cancer are both underway to investigate the efficacy of denosumab on systemic immunity and the local immunologic microenvironment (ISS 20,177,041).

It is likely that inhibition of RANK/RANKL can affect the immune response and how cancer cells respond to immune stimuli [227]. Since RANK/RANKL signaling is required to NF-κB activation, this may help to clarify the relationship between the NF-κB pathway and activation of immune checkpoints in cancer. Future studies could seek to understand the mechanism of NF-κB activation through RANK signaling and its interaction with a variety of immune checkpoints [228, 229].

## Immunotherapy and anti-NF-κB agents

The undeniable role of NF-κB in cancer has led to it being proposed as an anti-cancer therapeutic target in many studies. Proteosome inhibition, IKK inhibition, inhibition of NF-κB translocation to the nucleus, and inhibition of its binding to DNA, are some approaches that have been studied to suppress the NF-κB pathway in recent research [229, 230]. However, because NF-κB is also involved in the innate and adaptive immune responses, in many cases, NF-κB pathway inhibitors utilizing could lead to side effects such as immunosuppression [231].

In order to minimize the systemic toxicity of anti-NF-κB compounds in the body, new approaches seek to target NF-κB by specifically inhibiting subunits, or only inhibiting them in specific cells. As mentioned earlier, the arrangement of NF-κB subunits and their concentration [232] has an effect on the function of this protein. Therefore, designing drugs to target specific subunits responsible for cancer promotion in NF-κB could be more satisfactory. On the other hand, NF-κB functions employ a wide range of mechanisms specifically dependent on cell type. In this case, specific targeting of NF-κB in particular cells that affect tumor progression could enhance the effectiveness of anti-cancer therapy [233, 234]. ICIs, especially anti-PD-L1/PD-1 signaling inhibitors, disrupt immune checkpoints in the tumor microenvironment, thus inhibiting T cell suppression and enhancing antitumor immune response. However, the fact that some patients do not respond to ICIs, along with their possible severe side effects, has led to more research into combination approaches to cancer treatment. Since NF-κB signaling and immune checkpoints are related, and in particular, the fact that NF-κB can regulate PD-L1 expression at transcriptional and post-transcriptional levels, or participate in the expression of ICOS, a combination approach with NF-κB inhibitors and ICIs could provide a new strategy to increase patient response rates. This is important because NF-κB has a critical contribution in the resistance of various cancers to conventional therapies, such as chemotherapy and radiotherapy [235]. Since the implementation of ICI in clinical settings, the issue of resistance to ICI due to interferon (IFN)-related mechanisms has posed a substantial challenge in enhancing patient survival. Despite extensive research efforts, the identification of specific Interferon-Stimulated Genes (ISGs) responsible for conferring resistance in cancer cells has remained elusive [94]. In their recent study, Cucolo and colleagues have taken a comprehensive, innovative, and rigorous approach to uncover the role of RIPK1 as a mediator of resistance to both extrinsic and intrinsic ICI. RIPK1 is found to exert a dual influence by promoting an immunosuppressive tumor microenvironment driven by myeloid cells through NF-κB activation and by redirecting TNFRSF-related signaling away from caspase-8-mediated cell death. Importantly, Cucolo et al. have elucidated that the scaffolding function of RIPK1 represents the key molecular target for neutralizing its pro-survival effects. This discovery introduces a promising clinical avenue for potential small molecule inhibition, offering a compelling strategy for reversing ICI resistance among cancer patients [95]. Moreover, Kawase et al. investigated the role of IFNγ signaling pathway defects in resistance to ICIs [236]. The researchers demonstrated that reduced MHC-I expression is the primary cause of resistance due to IFNγ signaling pathway defects, rather than the loss of inhibitory effects on cellular proliferation or decreased chemokine production. They found that chemokines recruiting effector T cells were mainly produced by immune cells, not cancer cells, in the TME of a mouse model with IFNγ signaling pathway defects. They also observed a response to ICIs in a patient with JAK-negative head and neck squamous cell carcinoma who maintained HLA-I expression levels. In addition, the study used CRISPR screening to identify molecules associated with elevated MHC-I expression independent of IFNγ signaling pathways. They found that guanine nucleotide-binding protein subunit gamma 4 (GNG4) maintained MHC-I expression via the NF-κB signaling pathway. The results suggest that patients with IFNγ signaling pathway defects are not always resistant to ICIs. The findings emphasize the importance of MHC-I expression among the pathways and the potential of NF-κB-targeted therapies to overcome such resistance [236]. Therefore, regulation of NF-κB could overcome the challenges of cancer treatment to some extent. Table 2 lists some studies on the combined inhibition of NF-κB and the blockade of immune checkpoints.

**Table 2:** Combining anti-NF-KB medicines with ICIs

Anti-NF-κBs	Activity	Combined to ICIs	Mechanism	Refs.
Curcumin (Curcuma longa derived polyphenol)	Inhibiting IKK activity	Inhibiting CSN5-associated kinase	Increasing in PD-1 ubiquitination Increasing in sensitive of inflammation-induced tumors to anti-CTLA4	[237]
Pentoxifylline (PTXF)	Inhibitor of c-Rel	Anti-PD-1 mAb leads to increasing in tumor infiltrating T cells number	c-Rel specificity	[68]
TNF-α Inhibitors	Inhibiting TNF-α (prophylactic blockade)	Anti-PD-1 Anti-CTLA-4	Macrophage depletion	[238]
Cyclooxygenase 2 Inhibitors Andrographolide Celecoxib	Inhibiting the transcriptional co-activator p300 Histone acetyltransferase's acetylation of NF-κB p50 Inhibit IKK activity	Anti-PD-1 mAb	Preventing NF-κB from attaching to the COX-2 promoter and inhibiting its transcription	[239–241]
EGFR-Tyrosine Kinase Inhibitors Gefitinib	Inhibiting NF-κB leads to decreasing in PD-L1	Anti-CTLA-4	Reduces PD-L1 expression	[125]
CDK4/6 Inhibitors	Inhibiting formation of Rb and thereby NF-κB p65	Anti-PD-L1 immunotherapy	Preventing binging of NF-κB to DNA and suppressing expression of PD-L1	[114, 242]
Denosumab	Anti-RANKL	Nivolumab + Ipilimumab	–	NCT03161756

## Conclusions

Immunotherapy has revolutionized the treatment of many cancers. The use of ICIs has become one of the most promising types of immunotherapy in recent years. Although most immunotherapy studies have focused on PD-L1 and CTLA4, many studies have shown that other immune checkpoints potential play an essential role in suppressing the immune response and allowing cancer progression. LAG3, for example, was found to act as an immune checkpoint in uveal melanoma, and its expression in these patients was significantly higher compared to other immune checkpoints. Therefore, selecting the best checkpoint to inhibit may be important for any specific cancer. For this reason, the present review attempted to address the relationship between the known immune checkpoints and the NF-κB pathway, although studies in this area are still limited.

However, the side effects caused by immune checkpoint inhibitors have limited their widespread clinical use, moreover, it has been reported that many patients do not respond to these agents. For instance, it has recently been shown that immunotherapy can be associated with cardiotoxic side effects. Autoimmunity has been documented in pembrolizumab-treated individuals and ipilimumab. Heart failure, heart fibrosis, and myocarditis have also been reported using anti-PD1 antibodies [243]. Interestingly, activation of NF-κB in cardiomyocytes is a known factor in heart failure and cardiomyopathy. Indeed, these effects are due to the activity of NF-κB to induce severe inflammatory responses and myocyte atrophy [244]. In such cases, it is possible that a combination of anti-NF-κB drugs and immune checkpoint inhibitors at the same time could further reduce side effects. In addition, the effects of NF-κB on the regulation of Treg cells, inducing inflammatory responses, and inhibiting T cells is becoming clearer. Although NF-κB can act as a tumor suppressor, in most cases, it promotes cancer progression in different tumors. Therefore, anti-NF-κB drugs, especially those that interfere with the PD-L1 and CTLA4 pathways, could be an excellent option to be combined with immune checkpoint inhibitors. This approach could also be helpful to overcome resistance to cancer treatments, such as chemotherapy and radiotherapy.

Bearing all the above information in mind, there are still many limitations to the use of anti-NF-κB agents. Their dual function in cancer and immune cells is one of these limitations. Because the activity of NF-κB in Treg cells leads to their proliferation and increases their ability to suppress the immune system, this acts in line with immune checkpoints. In contrast, as described above, immune checkpoints can block the NF-κB pathway, thereby preventing T cells from proliferating. Under these circumstances, it seems that a more detailed study of the mechanisms of action of immune checkpoints and their associated signaling pathways, particularly NF-κB, in specific cell types, is necessary for the development of improved immunotherapy approaches for cancer. Therefore, we emphasize once again the need for specific targeting of NF-κB in different types of cells, and the study of compounds that target specific subunits of NF-κB. The combination of anti-NF-κB agents and immune checkpoint inhibitors, although having great potential to treat various cancers, especially treatment resistant and rapidly progressing types, will still face many questions concerning mechanisms of action and possible adverse effects.

## Data Availability

Data sharing is not applicable to this article as no datasets were generated or analysed during the current study.
